# Febrile urinary-tract infection due to extended-spectrum beta-lactamase–producing Enterobacteriaceae in children: A French prospective multicenter study

**DOI:** 10.1371/journal.pone.0190910

**Published:** 2018-01-25

**Authors:** Fouad Madhi, Camille Jung, Sandra Timsit, Corinne Levy, Sandra Biscardi, Mathie Lorrot, Emmanuel Grimprel, Laure Hees, Irina Craiu, Aurelien Galerne, François Dubos, Emmanuel Cixous, Véronique Hentgen, Stéphane Béchet, on behalf of the Urinary-tract Infection due to Extended-Spectrum Beta-lactamase–producing Enterobacteriaceae in Children Group, Stéphane Bonacorsi, Robert Cohen

**Affiliations:** 1 Service de Pédiatrie Générale, Centre Hospitalier Intercommunal de Créteil, Créteil, France; 2 GPIP (Groupe de Pathologie Infectieuse Pédiatrique) de la SFP (Société Française de Pédiatrie), Paris, France; 3 Université Paris Est, IMRB-GRC GEMINI, Créteil, France; 4 Centre de Recherche Clinique (CRC), Centre Hospitalier Intercommunal de Créteil, Créteil, France; 5 Service des Urgences Pédiatriques, CHU Necker, Paris, France; 6 ACTIV, Association Clinique et Thérapeutique Infantile du Val de Marne, Saint-Maur des Fossés, France; 7 Service des Urgences Pédiatriques, Centre Hospitalier Intercommunal de Créteil, Créteil, France; 8 Service de Pédiatrie Générale, CHU Robert Debré, Paris, France; 9 Service de Pédiatrie Générale, CHU Trousseau, Paris, France; 10 Service des Urgences Pédiatriques, CHU Lyon, Lyon, France; 11 Service des Urgences Pédiatriques, CHU Bicêtre, Bicêtre, France; 12 Service des Urgences Pédiatriques, CHU Jean Verdier, Bondy, France; 13 Service des Urgences Pédiatriques, CHU Lille, Lille, France; 14 Service des Urgences Pédiatriques, Centre Hospitalier de Roubaix, Roubaix, France; 15 Service de Pédiatrie Générale, Centre Hospitalier de Versailles, Versailles, France; 16 Service de Microbiologie, Hopital Robert-Debré, AP-HP, Centre National de Référence associé *Escherichia coli*, Paris, France; 17 Unité Court Séjour, Petits Nourrissons, Service de Néonatologie, Centre Hospitalier Intercommunal de Créteil, Créteil, France; Chang Gung Memorial Hospital, TAIWAN

## Abstract

**Objectives:**

To assess the management of febrile urinary-tract infection (FUTIs) due to extended-spectrum β-lactamase–producing *Enterobacteriaceae* (ESBL-E) in children, the Pediatric Infectious Diseases Group of the French Pediatric Society set up an active surveillance network in pediatric centers across France in 2014.

**Materials and methods:**

We prospectively analysed data from 2014 to 2016 for all children < 18 years old who received antibiotic treatment for FUTI due to ESBL-E in 24 pediatric centers. Baseline demographic, clinical features, microbiological data and antimicrobials prescribed were collected.

**Results:**

301 children were enrolled in this study. The median age was 1 year (IQR 0.02–17.9) and 44.5% were male. These infections occurred in children with history of UTIs (27.3%) and urinary malformations (32.6%). Recent antibiotic use was the main associated factor for FUTIs due to ESBL-E, followed by a previous hospitalization and travel history. Before drug susceptibility testing (DST), third-generation cephalosporins (3GC) PO/IV were the most-prescribed antibiotics (75.5%). Only 13% and 24% of children received amikacine alone for empirical or definitive therapy, respectively, whereas 88.7% of children had isolates susceptible to amikacin. In all, 23.2% of children received carbapenems in empirical and/or definitive therapy. Cotrimoxazole (24.5%), ciprofloxacin (15.6%) and non-orthodox clavulanate–cefixime combination (31.3%) were the most frequently prescribed oral options after obtaining the DST. The time to apyrexia and length of hospital stay did not differ with or without effective empirical therapy.

**Conclusions:**

We believe that amikacin should increasingly take on a key role in the choice of definitive therapy of FUTI due to ESBL-E in children by avoiding the use of carbapenems.

## Introduction

Febrile urinary tract infections (FUTIs) are the most common proven bacterial infections in pediatric clinical practice. They can be associated with high morbidity and long-term complications such as renal scarring, hypertension, and chronic renal failure [1,2]. Early diagnosis and adequate treatment decrease the risk of renal scarring risk and other complications [3]. FUTIs are most frequently due to *Enterobacteriaceae*, mainly *Escherichia coli* [4,5].

The emergence of extended-spectrum β-lactamase–producing *Enterobacteriaceae* (ESBL-E) as a cause of FUTI presents a serious threat to public health because therapeutic options are limited [6,7]. A few years ago, ESBL-E were isolated mainly in hospital settings and other healthcare facilities. However, such organisms have spread in the community, and the incidence of community-onset FUTIs due to ESBL-E isolates has increased worldwide [8,9].

ESBL are enzymes that hydrolyze penicillins, cephalosporins but spare cephamycins (cefoxitin, cefotetan), moxalactam and carbapenems and are mostly produced by *Enterobacteriaceae*. Some clones of *E*. *coli*, including the Sequence Type (ST) 131 and more recently the ST410, have emerged in recent years by pandemics [10,11].

International guidelines emphasize oral antibiotics as first-line treatment of FUTIs in children [12,13]. However, no oral antibiotic as first-line treatment is regularly active against ESBL-E and there are few intravenous options. The standard treatment for severe infections due to ESBL-E remains carbapenems. However, the uncontrolled use of carbapenems in several countries has led to the emergence of carbapenemase producing *Enterobacteriacae* (*Klebsiella pneumoniae* carbapenemase and New Delhi metallo-beta-lactamase 1 (NDM-1), in particular), which are sometimes resistant to all known antibiotics [14]. Furthermore, the recent changes including colistin resistance by carriage of the mobilized colistin resistance (*mcr1*) gene are increasingly worrisome [15]. Less than 50% of patients with ESBL-E related infections were de-escalated after empirical treatment with carbapenems [16]. Saving carbapenems when alternative treatment exists is one of the therapeutic challenges. The recent French guidelines took into account both the increase in ESBL-E strains among those isolated from FUTIs, the need to spare the carbapenems and recommend the use of amikacin as an alternative first-line treatment [17]. However these recommendations were not based on strong evidence such as prospective multicenter pediatric cohort or randomized controlled trials, which have led to various therapeutic attitudes among centers.

To assess the management of FUTIs due to ESBL-E infection in children, the Pediatric Infectious Diseases Group (GPIP/ACTIV) of the French Pediatric Society (SFP) set up an active surveillance network in pediatric centers across France in 2014. The aim of our study was to describe the clinical and microbiological spectrum, antibiotic choice and clinical outcome of FUTIs due to ESBL-E over a 3-year period.

## Materials and methods

### Population

The National Observatory of FUTI due to ESBL-E in children was created by the GPIP/ACTIV network involving 24 pediatric centers (pediatric and emergency departments) and their microbiology departments. Throughout France, 6 regions (Ile de France, Hauts-de-France, Pays de la Loire, Auvergne-Rhône-Alpes, Normandie, Provence-Alpes-Côte d'Azur) were involved. Between March 2014 and March 2017, all children with FUTI due to ESBL-E were enrolled in this prospective observational study. For this hospital-based active surveillance, a clinical investigator in each participating ward completed a standardized data form, which was sent by electronic or postal mail to the investigating center (GPIP/ACTIV). A scientific committee validated all data.

The following diagnostic criteria has been used for all inpatients or outpatients less than 18 years old who had fever, clinical signs associated with positive ESBL-E infection in urine culture and antibiotic treatment targeting this strain. From this cohort, we selected a first group of patients with FUTI defined by strict adherence to French recommendations concerning urine collection methods [18], including when the urine was collected by bag: leukocyturia ≥10^4^/mL and positive culture ≥10^5^ colony forming units (CFUs). We selected a second group of patients with FUTI defined according to guidelines of the European Association of Urology and European Society for Pediatric Urology (EAU/ESPU) [19], with one of the following positive test results: positive culture ≥10^4^ CFU obtained by mid-stream urine sample or ≥10^3^ CFU obtained by urethral catheterization or any level of positive culture obtained by suprapubic puncture. The third selected group was patients with FUTI defined according to guidelines of the American Academy of Pediatrics (AAP) [13], with positive test results for one of the following: positive culture ≥5.10^4^ CFU obtained by urethral catheterization or ≥5.10^4^ CFU obtained by suprapubic puncture.

Children with asymptomatic bacteriuria or mixed microbial strains were excluded. The first isolate from each patient was studied and we excluded repeated episodes.

### Definitions

Empirical therapy (ET) was defined as antimicrobial therapy applied before drug susceptibility testing (DST) results became available. Definitive therapy (DT) was defined as antibiotics given after DST. Effective empirical treatment (EET) involved at least one active drug *in vitro* against the strain with at least 48-hr including gentamicin, amikacin, piperacillin-tazobactam, ceftazidime, ciprofloxacin, ofloxacin, imipenem, meropenem, ertapenem, and cotrimoxazole. Ineffective empirical treatment (IET) was considered if the microorganism was resistant to the administered antibiotic. Treatment success was defined as apyrexia within 5 days with no local complications such as abscess and no recurrence within 10 days.

### Data collected

Data were collected on date of birth, gender, anamnesis, medical and surgical history including congenital anomalies of the kidney and urinary tract (CAKUT), risk factors for carriage or infection of ESBL-E [20,21], clinical signs, biological factors (C-reactive protein [CRP] and procalcitonin levels) and urine microbiological data (urine collection method, dipstick, leukocyturia, urine Gram staining and culture results, blood culture and DST). Data on ET, DT, time to apyrexia, length of hospital stay (LOS) and clinical outcomes were also collected.

### Microbiology

Each center (microbiology laboratory) had to provide the frequency of ESBL-E strains among the *Enterobacteriaceae* isolated in urine for the 3 study periods: March 2014-March 2015, March 2015-March 2016 and March 2016–2017. ESBL-E was identified by standard methods in the microbiology laboratory of each hospital as recommended by the antibiogram committee of the French Society of Microbiology [22]. In brief, one colony of each morphologic type growing on the medium was identified by using the API20E system (bioMérieux, Marcy l’Etoile, France) or with the Bruker Biotyper Matrix-Assisted Laser Desorption Ionization–Time of Flight Mass Spectrometer. Antibiotic susceptibility was determined by using the disc diffusion method on Mueller-Hinton agar and interpreted as specified by the European Committee on Antimicrobial Susceptibility Testing (http://www.eucast.org/). Possible ESBL production was defined as synergy between clavulanic acid and at least one of the extended-spectrum cephalosporins (ceftazidime, cefotaxime, or cefepime) or aztreonam [23]. For several laboratories, the minimum inhibitory concentration (MIC) of cefixime and amoxicillin-clavulanate (AC) was determined by the Etest method (AB bioMérieux, Solna, Sweden) and for other laboratories, the Etest was also used to evaluate the activity of the antimicrobial combination of cefixime and AC as previously described [24]. The MIC of the combination was interpreted as the value at which the inhibition zone intersected the scale on the Etest strip. Synergy was evaluated by calculating the fractional inhibitory concentration (IC) index.

### Ethics approval

The data collection was approved by the French National Data Protection Commission (CNIL, no. 913582), the Committee on the Processing of Research Information (CCTIRS, no. 13.341) and the Créteil Intercommunal Hospital Ethics Committee. All legal guardians of included children provided oral informed consent. The study was registered at ClinicalTrials.gov (registration no. NCT02832258).

### Statistical analysis

Data were entered by using an electronic database (PHP/MySQL) and analyzed by using Stata/SE 13.0 (StataCorp, College Station, TX, USA). Quantitative data were analyzed by means, standard deviations and medians, and categorical data by frequencies and percentages. First and second lines of antibiotic therapies were analyzed. The time to apyrexia and length of hospital stay were drawn on Kaplan-Meier curves and compared using the log-rank test. P<0.05 were retained as significant.

## Results

### Frequency of ESBL-E

**Fig 1:**
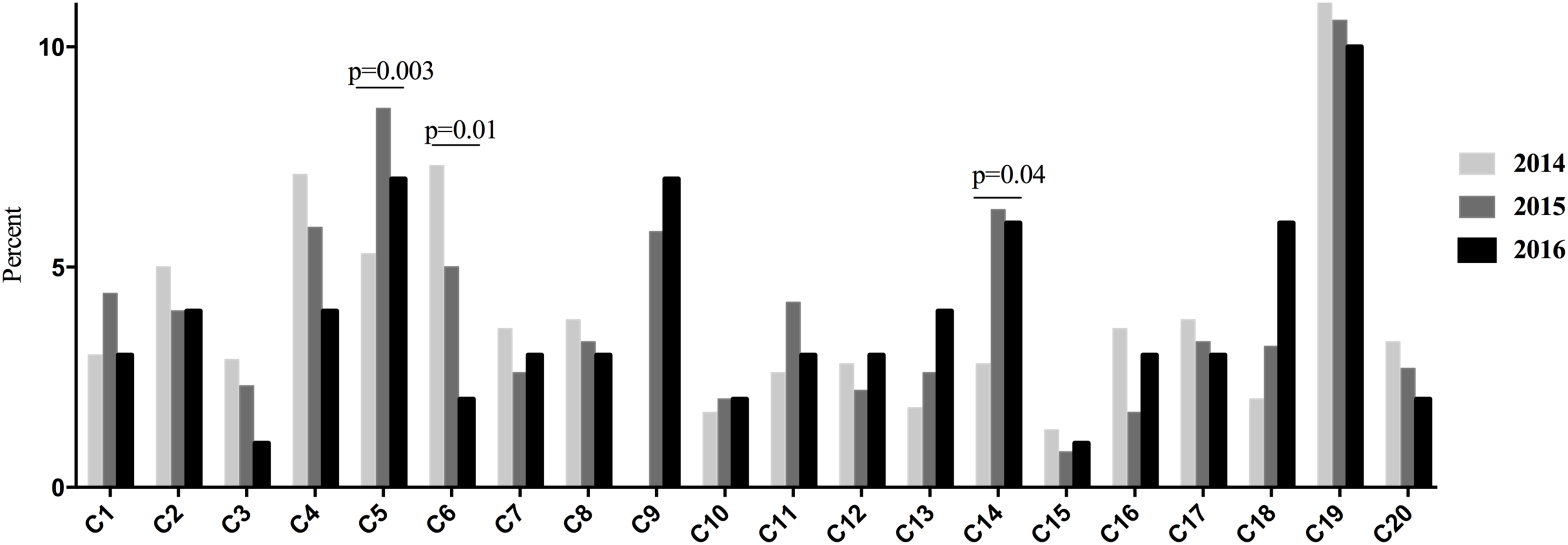
Frequency of ESBL-producing Enterobacteriaceae in urine in each microbiology center between 2014 and 2016. C, center.

The frequency of ESBL-E strains isolated in urine in the 20 microbiology centers ranged from 0.8% to 10% per year over a 3-years period. There was no significant variation over time except in 3 centers (Fig 1).

### Demographic and epidemiologic data

In the 3-year study period, 301 children were enrolled, including 283, 151 and 87 FUTI according to the French recommendations, EAU/ESPU and AAP guidelines, respectively. With FUTI according to French recommendations, the median age was 1 year (min-max: 0.02–16.3) and according to the EAU/ESPU guideline, 2.14 years (min-max: 0.02–16.3). With FUTI according to French, EAU/ESPU and AAP guidelines, 44.2%%, 28.5% and 30% of patients, were males respectively. Table 1 summarizes the demographic and epidemiologic characteristics of the study population.

**Table 1:** Epidemiology and clinical characteristics of patients with febrile urinary-tract infections (FUTIs).

Parameters	Study population n = 301	FUTI by French recommendations n = 283	FUTI by EAU/ESPU guidelines n = 151	FUTI by AAP guidelines n = 87
**Age, years, mean (±SD)** ** median [min-max]**	2.6 (3.7) 1 [0.02–17.9]	2.5 (3.6) 1 [0.02–16.3]	3.9 (4.4) 2.14 [0.02–16.3]	2.4 (3.3) 1 [0.02–15.9]
**Sex male**	134 (44.5%)	125 (44.2%)	43 (28.5%)	26 (30%)
**Recurrent UTI**	82 (27.3%)	74 (26.2)	50 (33.3%)	23 (26.7%)
**Vesicoureteral reflux (VUR)**	31 (10.3%)	30 (10.6%)	21 (14%)	10 (11.6%)
**Other CAKUT (excluding VUR)**	67 (22.3%)	61 (21.6%)	36 (23.8%)	25 (28.7%)
**Underling disease**	47 (15.7%)	43 (15.2%)	28 (18.7%)	15 (17.5%)
**Use of antibiotics in the previous 3 months**	116 (40.8%)	108 (40.5%)	62 (44.3%)	34 (42%)
**Antibiotic prophylaxis**	42 (14%)	41 (14.6%)	22 (14.8%)	12 (13.9)
**Surgery in the previous year**	39 (13.3%)	38 (13.8%)	26 (17.7%)	18 (21.4%)
**Hospitalization in the previous year**^a^	110 (37.3%)	104 (37.5%)	53 (35.8%)	34 (39.5%)
**Travel in a foreign country in the previous year**	84 (31%)	81 (31.4%)	50 (37%)	33 (40.7%)
**Surgery in foreign country**	3 (1%)	3 (1.1%)	3 (2.1%)	2 (2.3%)
**Contact with a person of the entourage (living under the same roof) hospitalized or travelling in the previous 6 months**	84 (38.2%)	82 (39%)	45 (42.4%)	27 (38.6%)

^a^ Hospitalization for any medical or surgical reason in a care unit at the hospital

Three urinary malformations were common regardless of classification: pyelocaliceal and/or ureteral dilatation, ureteropelvic junction obstruction and duplex kidney (Table 2).

**Table 2:** Urinary-tract abnormalities of the study population.

Abnormalities, n (%)	Study population n = 301	FUTI by French recommendations n = 283	FUTI by EAU/ESPU guidelines n = 151	FUTI by AAP guidelines n = 87
**Vesicoureteral reflux**	31 (10.3%)	30 (10.6%)	21 (14%)	10 (11.6%)
**Pyelocalyceal and/or ureteral dilatation**	20 (6.6%)^a^	18 (6.4%)^a^	5 (3.3%)	4 (4.6%)
**Duplex kidney**	11 (3.6%)^a^	10 (3.5%)^a^	6 (4%)	3 (3.4%)
**Ureteropelvic junction obstruction**	10 (3.3%)	10 (3.5%)	7 (4.6%)	1 (1.1)
**Hypospadias**	5 (1.7%)^a^	4 (1.4%)^a^	2 (1.3%)	2 (2.3%)
**Bladder exstrophy**	4 (1.3%)^a^	3 (1.1%)^a^	1 (0.7%)	2 (2.3%)
**Posterior urethral valves**	3 (1%)^a^	3 (1.1%)^a^	1 (0.7%)	-
**Complex urinary malformation**	3 (1%)^a^	2 (0.7%)^a^	1 (0.7%)	-
**Multicystic dysplastic kidney**	3 (1%)^a^	3 (1.1%)^a^	2 (1.3%)	2 (2.3%)
**Ectopic kidney**	3 (1%)	3 (1.1%)	2 (1.3%)	1 (1.1%)
**Megaureter**	2 (0.7%)^a^	2 (0.7%)^a^	2 (1.3%)	2 (2.3%)
**Neurogenic bladder**	2 (0.7%)^a^	2 (0.7%)^a^	2 (1.3%)	2 (2.3%)
**Hydronephrosis**	2 (0.7%)	2 (0.7%)	1 (0.7%)	-
**Horseshoe kidney**	2 (0.7%)^a^	2 (0.7%)^a^	1 (0.7%)	1 (1.1%)
**Renal hypodysplasia**	2 (0.7%)	2 (0.7%)	1 (0.7%)	-
**Multiple bladder diverticula**	1 (0.3%)^a^	1 (0.3%)^a^	1 (0.7%)	1 (1.1%)
**Megacystis**	1 (0.3%)	1 (0.3%)	1 (0.7%)	1 (1.1%)
**Intravesical ureterocele**	1 (0.3%)^a^	1 (0.3%)^a^	1 (0.7%)	1 (1.1%)
**Ectopic ureter-bladder**	1 (0.3%)^a^	1 (0.3%)^a^	1 (0.7%)	1 (1.1%)
**Renal agenesis**	1 (0.3%)^a^	1 (0.3%)^a^	-	-

^a^Associations: 1 Pyelocalyceal and/or ureteral dilatation + duplex kidney; 1 Pyelocalyceal and/or ureteral dilatation + posterior urethral valves; 1 Pyelocalyceal and/or ureteral dilatation + bladder exstrophy; 1 Pyelocalyceal and/or ureteral dilatation + hypospadias; 1 Duplex kidney + multicystic dysplastic kidney; 1 Duplex kidney + intravesical ureterocele; 1 Megaureter + multiple bladder diverticula; 1 Bladder exstrophy + ectopic ureter-bladder; 1 Megaureter + multiple bladder diverticula; 1 Renal agenesis + horseshoe kidney; 1 Complex urinary malformation + hypospadias

### Clinical and microbiological data

Overall, 58% of this cohort was hospitalized. Hemodynamic disorders were present in 7.8%, 4.6% and 3.4% of FUTI cases according to French, EAU/ESPU and AAP guidelines, respectively. The median temperature was 39.4 (min-max: 38–42.1) according to the French recommendation and was comparable with the other guidelines. The median CRP and procalcitonin levels were 75 mg/L (min-max: 1–404) and 1 ng/mL (min-max: 0–110) according to the French recommendation and were comparable with other guidelines. Overall, blood cultures were performed in 175 children (58.1%): seven children were diagnosed with bacteremia (4%), five were less than 4 months old. The pathogens were *E coli* (6 cases) and *K pneumoniae* (1 case). Table 3 summarizes the microbiological data for the study population.

**Table 3:** Microbial characteristics of the study population.

Parameters	Study Population n = 301	FUTI by French recommendations n = 283	FUTI by EAU/ESPU guidelines n = 151	FUTI by AAP guidelines n = 87
**The method of urine collection**				
*Urine bag*	139 (46.2%)	130 (46%)	-	-
*Midstream*	70 (23.2%)	66 (23.3%)	66 (43.7%)	-
*Urethral catheterization*	88 (29.2%)	86 (30.4%)	84 (55.6%)	86 (99%)
*Suprapubic aspiration*	1 (0.4%)	1 (0.3%)	1 (0.7%)	1 (1%)
*Missing data*	3 (1%)	-	-	-
**Positive culture**				
*Escherichia coli*	264 (87.8%)	249 (88%)	135 (89.4%)	78 (89.7%)
*Klebsiella pneumonia*	32 (10.6%)	29 (10.1%)	15 (9.9%)	9 (10.3%)
*Enterobacter cloacae*	2 (0.7%)	2 (0.7%)	1 (0.7%)	-
*Cedecea sp*.	1 (0.3%)	1 (0.4%)	-	-
*Klebsiella oxytoca*	1 (0.3%)	1 (0.4%)	-	-
*Citrobacter koseri*	1 (0.3%)	1 (0.4%)	-	-

The DST results were comparable between the groups whatever the guideline (Table 4).

**Table 4:** Susceptibility profile of *Enterobacteriaceae* isolates in groups of children with FUTI due to ESBL-E.

Susceptible agents	Study Population n = 301	FUTI by French recommendations n = 283	FUTI by EAU/ESPU guidelines n = 151	FUTI by AAP guidelines n = 87
Ampicillin/amoxicillin	0 (0%)	0 (0%)	0 (0%)	0 (0%)
Amoxicillin/clavulanic acid	72 (24%)	67 (23.7%)	39 (26%)	26 (29.9%)
Piperacillin-tazobactam	207 (74.7%)	196 (75.1%)	100 (76.3%)	52 (73.2%)
Cefotaxime/ceftriaxone	5 (1.7%)	4 (1.4%)	4 (2.7%)	2 (2.4%)
Ceftazidime	49 (16.5%)	46 (16.5%)	24 (16.2%)	15 (17.8%)
Cefixime	3 (1.1%)	3 (1.2%)	3 (2.3%)	2 (2.6%)
Cefepime	29 (13.2%)	27 (13.2%)	15 (13.2%)	10 (13.9%)
Ertapenem	287 (98.6%)	270 (98.5%)	144 (97.9%)	85 (98.8%)
Imipenem	259 (99.6%)	244 (99.6%)	136 (99.3)	80 (100%)
Gentamicin	180 (60%)	167 (59.2%)	90 (60%)	49 (57%)
Amikacin	266 (88.7%)	250 (88.6%)	134 (89.3%)	76 (88.4%)
Cotrimoxazole	85 (29.6%)	80 (29.6%)	37 (25.8%)	27 (33.3%)
Nalixid acid	99 (34.8%)	91 (34.2%)	41 (29.1%)	26 (31.7%)
Ciprofloxacin	149 (51%)	139 (50.7%)	70 (47.6%)	42 (50%)
Nitrofurantoin	218 (92.8%)	202 (92.2%)	104 (93.7%)	48 (94.1%)
Fosfomycin	196 (97.5%)	187 (97.9%)	92 (96.8%)	44 (95.6%)

Overall, 88.7% of patients had isolates susceptible to amikacin, 60% to gentamicin and 74.7% to piperacillin-tazobactam. Among treatment options for oral antibiotic relay, 29.6% of isolates were susceptible to cotrimoxazole and 51% to ciprofloxacin, whereas 37% of isolates were resistant to both these antibiotics. For the non-orthodox AC-cefixime combination, among 99 strains tested, 62.4% had MIC ≤0.5 mg/L and 91% ≤1 mg/L.

### Treatment and outcomes

#### FUTI according to French recommendations

In empirical treatment, oral/intravenous 3GC was the most-used antibiotic therapy, in monotherapy (35.3% of cases) or associated with aminoglycosides (gentamicin or amikacin) in 40.2% of cases. Amikacin alone and carbapenems were used for 13% and 4.3% of children, respectively. The antibiotic therapy was modified in 275 (91.4%) FUTI cases after DST results were obtained. In DT, patients received amikacin alone (24%), carbapenems with or without aminoglycosides (18.6%), piperacillin-tazobactam or piperacillin-tazobactam-aminoglycosides (3.6%). In oral antibiotic relay, cotrimoxazole and quinolone were the most frequently used antibiotic therapy for 24.5% and 15.6% of children, respectively. The non-orthodox AC-cefixime combination was given to 86 children (31.3%). The distribution of antibiotic therapy was similar whatever the guideline applied. Overall, 97.3% of cases had an existing effective treatment other than carbapenems, and only 1.3% had an existing effective treatment other than carbapenems without the option of oral treatment (resistant to cotrimoxazole, ciprofloxacin/nalidixic acid and AC-cefixime combination ≥1 mg/L MIC).

**Fig 2:**
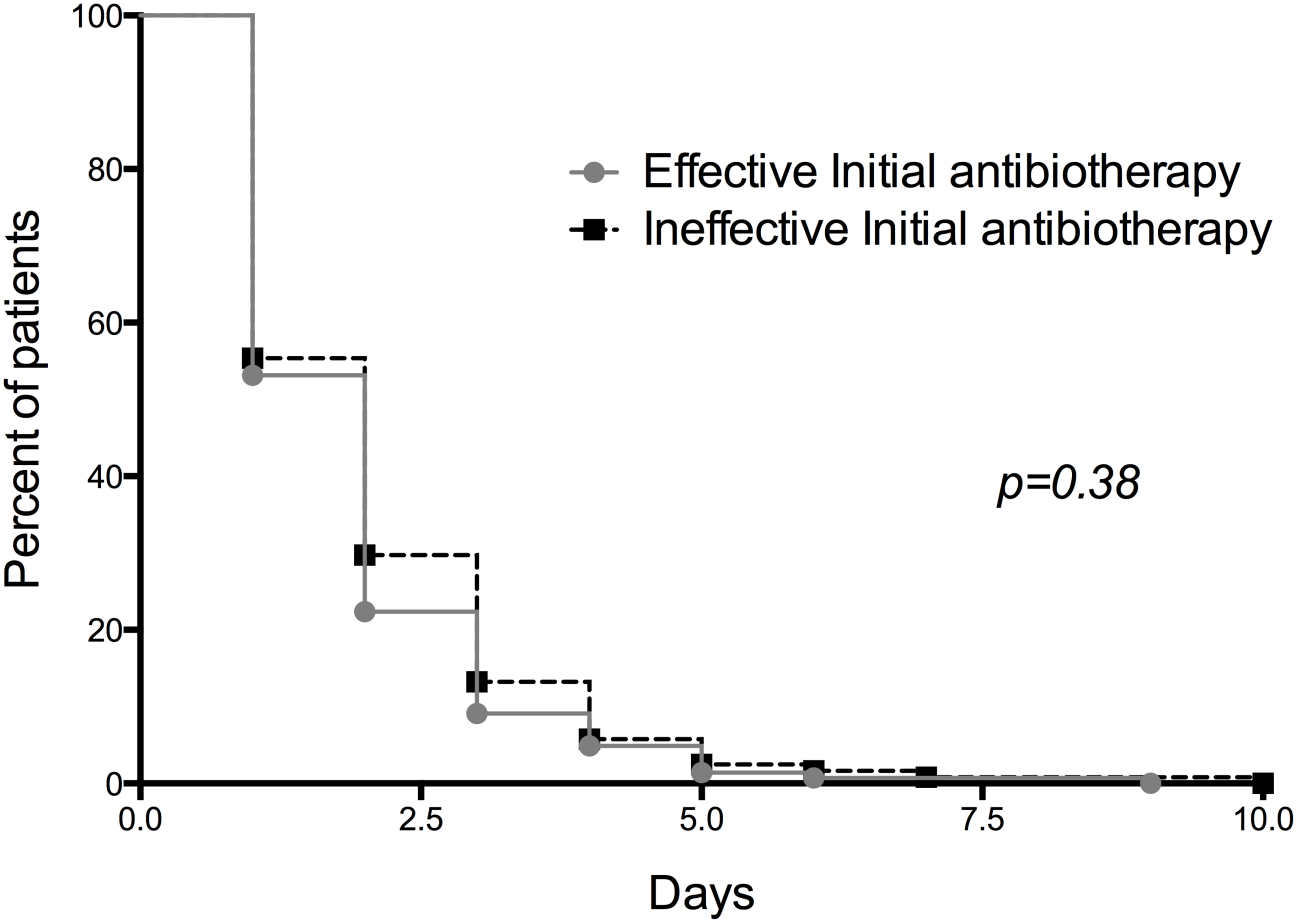
Time to apyrexia. Kaplan-Meier estimates of time to apyrexia in patients with effective and ineffective empirical treatment.

**Fig 3:**
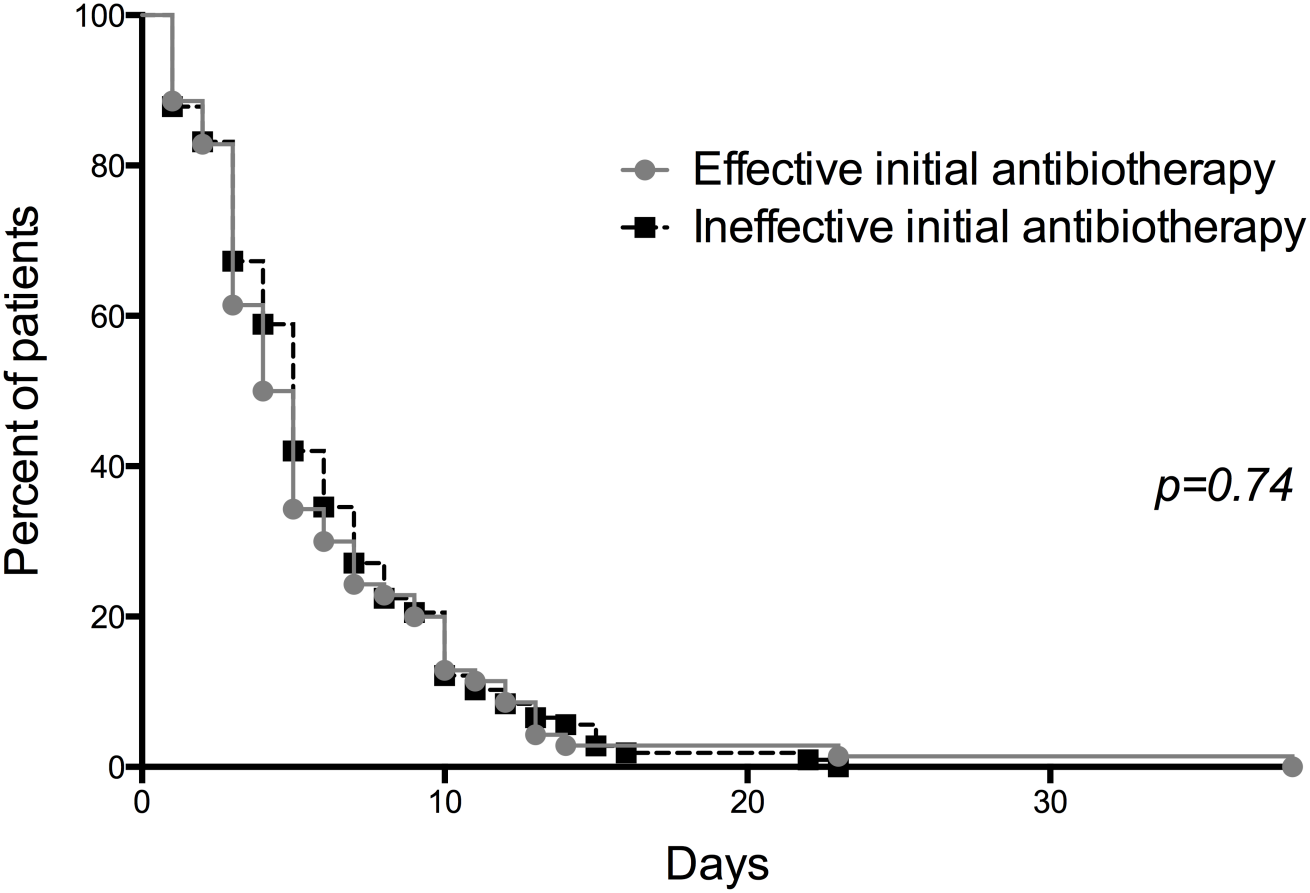
Length of hospital stay. Kaplan-Meier estimates of length of hospital stay in patients with effective and ineffective empirical treatment.

Overall, the time to apyrexia was 1.8 days (min-max: 0–10) and the median hospital stay was 3.4 days (SD 4.6) (min-max: 0–38). These last two features were similar whatever the guideline applied. The time to apyrexia did not differ with EET or IET (log rank p = 0.38) (Fig 2). Similarly, LOS did not differ with EET or IET (log rank p = 0.74) (Fig 3).

In hospitalized patients, the LOS was higher for those receiving carbapenems (111 patients receiving amikacin vs 39 patients receiving carbapenems without amikacin, log-rank: p <0.0001). The time to apyrexia was similar in both these groups (163 patients receiving amikacin vs 43 patients receiving carbapenems without amikacin, log-rank: p = 0.16).

Moreover, LOS and time to apyrexia did not differ in patients with or without urinary malformation (log-rank: p = 0.71 and p = 0.73, respectively). The frequencies of ET and DT with carbapenems did not significantly differ in patients with or without urinary malformations (3.6% vs 3.3%, p = 0.3, and 18.8% vs 27.8%, p = 0.06, respectively). Overall, 95% of children were apyretic in less than 5 days, with no local complications such as abscess and no recurrence within 10 days.

## Discussion

Our study suggests that amikacin should be considered a key drug for DT of FUTIs due to ESBL-E in children to reduce the prescription of carbapenems. It is well known that carbapenems are considered the most reliable treatment for infections caused by ESBL-producing bacteria [6]. Despite their utility, resistance has emerged, which has led to finding alternative antibiotics for UTIs so that carbapenems can be reserved for more serious infections. Only 24% children in our cohort received amikacin as DT whereas about 90% of bacterial isolates in our study were susceptible to amikacin. Furthermore, amikacin can be administrated as a daily single dose and allows outpatient care. Moreover, pharmacokinetics and pharmacodynamics (PK/PD) of aminoglycosides, in particular their weak bile and digestive elimination suggest a lower impact on intestinal microbiota [25]. Han et al. recently described their positive retrospective experience with aminoglygosides in UTIs due to ESBL-E [26]. Our team also recently described the interest of amikacin administered once a day for FUTIs related to *Enterobacteriaciae* infection with or without an ESBL-producing resistance mechanism [27]. Certainly, the daily administration with 30-min intravenous injection less than 5 days (usually between 2 and 3 days) has been shown to promote tissue diffusion and renal concentration while limiting renal and hearing toxicity [28]. In our study, no side effects were reported. For these reasons, we believe that amikacin is a major therapeutic solution to treat FUTI in first line or in DT for FUTI due to ESBL-E.

Another treatment option is piperacillin-tazobactam. Bouchillon et al. showed piperacillin-tazobactam susceptibility among urinary isolates from hospitalized US patients to be 81.7% for ESBL-*E*. *coli* and 31.3% for ESBL-*K*. *Pneumonia* [29]. Piperacillin-tazobactam is also largely eliminated by the kidney, with 68% of piperacillin and 80% of tazobactam excreted in urine as unchanged drugs [30]. Despite a paucity of data for the use of piperacillin-tazobactam for UTIs due to ESBL-E, the evidence seems favourable, because a recent study showed activity at least equal to carbapenems in bacteremia due to ESBL-E [31].

Temocillin is now an additional option but was not authorized during the study period in France. This ß-lactam compound shows time-dependent activity, strong protein binding and renal tubular excretion [32]. Furthermore, temocillin activity in a mouse model of UTI due or not to ESBL-E was similar, with MIC of the strains ≤16 mg/L [33]. However, two intravenous doses per day is mandatory, which leads to difficulties with ambulatory treatment.

Despite their high rate of susceptibility, nitrofurantoin and fosfomycin are commonly used to treat cystitis in adults [34,35]. There are no data on their efficiency in FUTI in children Moreover, the tolerance of nitrofurantoin needs to be investigated more in children.

Ceftolozane-tazobactam and ceftazidime-avibactam are recent compounds with little clinical experience and should be reserved for specific situations, in particular infections related to resistant *Pseudomonas aeruginosa* and carbapenemase producing *Enterobacteriacae* except NDM-1 [36,37].

After DST, oral relay can be used in one fifth of cases with cotrimoxazole and one half with quinolones. Most remaining strains (62.4% and 91%) were susceptible to the non-orthodox AC-cefixime combination, with MIC_50_ and MIC_90_ for cefixime at 0.5 and 1 mg/l, respectively. This combination shows interesting *in vitro* activity, with some clinical reports of efficacy [38,39]. We believe these three compounds should be considered for oral relay of FUTIs due to ESBL-E in children.

Additionally, we showed that despite initial inappropriate treatment, FUTIs have similar outcomes in time to apyrexia and LOS as with appropriate treatment. Indeed, Greenhouse et al. recently demonstrated in a retrospective observational study of UTIs due to ESBL-E that inappropriate empirical and definitive antimicrobial therapy were associated with short-term clinical improvement [40]. Several explanations could put forward. First, some patients may not have had true FUTI. However, when we applied the EAU/ESPU and AAP diagnostic criteria, we found the same results. Second, the concentration of antibiotics is probably sufficient in urine, blood, and in renal parenchyma to resolve FUTIs due to ESBL-E. Finally, the infection may spontaneously resolve. Indeed, the renal bacterial burden, interleukin 6 concentration, and histological inflammatory lesions did not significantly differ in mice with ESBL-E infection with and without appropriate treatment [41].

The susceptibility pattern of isolated ESBL-E in our cohort was similar to that found in hospitals from Northwest England and North Wales between 2007 and 2012 [42]. In the Study for Monitoring Antimicrobial Resistance Trends (SMART) in Canada and the United States, susceptibility to amikacin remained high, between 95.4% and 100% [43]. This variability in antimicrobial susceptibility patterns among E-ESBLs between studies depends on local epidemiology according to the levels and types of plasmid-mediated resistance genes [44]. Similar to a few other studies, we found a higher rate of FUTI due to ESBL-E among female children [45,46]. According to previous studies and case series [47,48], ESBL-E infections affected mainly children with urinary malformations (vesicoureteral reflux and other CACKUT), history of UTI, recent antibiotic use (especially penicillin and cephalosporin’s), previous hospitalization and travel history. Strikingly, only 24.9% of children had risk factors.

Although in our study, urine collection for diagnosis of UTIs in France mainly involved a urine bag despite national and international recommendations [12,13,17]; this collection represents and reflects the current practice in many countries. When selecting patients according to European and US guidelines where the probability of having a real acute pyelonephritis is highest, we found comparable results. Although the presence of structural renal damage from 99mTc dimercaptosuccinic acid (DMSA) scans performed during the FUTI episode, is the golden standard for diagnosis of acute pyelonephritis, it is a difficult exam to obtain in common practice.

Our study has several limitations. First, we did not perform DMSA scanning during the febrile episode (to ensure the diagnosis of pyelonephritis) or during follow-up (to assess the ratio of renal scars). Second, we did not have a control group of patients with FUTIs due to non–ESBL-E strains. Finally, if we consider patients sampled only by urethral catheterization or suprapubic puncture, the number of assessable patients represents 30% of this cohort. However, 76% of our patients not only had substantial bacteriuria but also pyuria and high levels of CRP and/or procalcitonin (CRP ≥60 mg/L and/or ≥0.5 ng/mL) suggesting a high probability of renal involvement [49].

## Conclusion

We believe that amikacin should increasingly take on a key role in the choice of definitive therapy of FUTI due to ESBL-E in children by avoiding the use of carbapenems. Depending on the susceptibility of isolated strains, different oral relay possibilities were available: 30% of isolates were susceptible to cotrimoxazole, 50% were susceptible to ciprofloxacine and only 37% were resistant to both antibiotics, which led to the prescription of a non-orthodox combination.
